# Vaccine potential of LenA and LcpA proteins of *Leptospira interrogans* in combination with *Escherichia coli* heat-labile enterotoxin, B subunit (LTB)

**Published:** 2019-02

**Authors:** Mehran Ghazali-Bina, Mohammad Reza Pourmand, Abbas Mirshafiey, Ronak Bakhtiari, Azad Khaledi, Hamid Kazemian, Davoud Afshar, Muhammad Ibrahim Getso, Saeid Eshraghi

**Affiliations:** 1 Department of Microbiology, School of Public Health, Tehran University of Medical Sciences, Tehran, Iran; 2 Department of Immunology, School of Public Health, Tehran University of Medical Sciences, Tehran, Iran; 3 Infectious Diseases Research Center, Kashan University of Medical Sciences, Kashan, Iran; 4 Department of Microbiology and Immunology, Faculty of Medicine, Kashan University of Medical Sciences, Kashan, Iran; 5 Department of Microbiology and Virology, School of Medicine, Zanjan University of Medical Sciences, Zanjan, Iran; 6 Department of Medical Mycology, School of Public Health, Tehran University of Medical Sciences, Intenational College, Tehran, Iran

**Keywords:** Immunogenicity, *Leptospira interrogans*, Recombinant proteins, Vaccine

## Abstract

**Background and Objectives:**

Leptospirosis is a zooanthroponosis caused by the genus of *Leptospira*. It is an emerging public health problem due to its increasing incidence. The achievement to a vaccine that prevent from entrance of *Leptospira interrogans* to the deeper tissues of the host is needed. This study aimed to investigate the immunogenicity of LcpA (rLcpA) and LenA (rLenA) recombinant proteins in combination with LTB (rLTB) recombinant protein as an adjuvant against leptospiral infection in hamsters.

**Materials and Methods:**

The genes encoding these proteins were cloned into pGH cloning vector and then *lenA, lcpA* and *ltb* genes subcloned into pET-15b and pET-28a expression vectors, respectively. The hamsters were immunized with the purified recombinant proteins and challenged with *Leptospira interrogans* for evaluation of their survival. The antibody responses to the recombinant proteins were determined by ELISA. Then, data entered into SPSS software. Statistical Kruskal–Wallis test was used to compare the significant differences among different groups. The groups with significant differences were further analyzed by post hoc tests. The *p* value < 0.05 statistically was considered significant.

**Results:**

Immunized hamsters with rLenA-plus-rLTB, rLcpA-plus-rLTB and rLenA-plus-rLcpA-plus-rLTB proteins showed 60%, 74%, and 80% survival rates, respectively. A significant amount of interleukin-17 (IL-17), interleukin-4 (IL-4) and gamma interferon (IFNγ) cytokines were produced in immunized hamsters.

**Conclusion:**

Based on our findings, rLcpA and rLenA proteins in combination with rLTB can protect the hamsters against *L. interrogans* and effectively induce a protective antibody response. Thus, these proteins can be used as an additional prophylactic tool against leptospira.

## INTRODUCTION

Leptospirosis is an emerging global zoonotic disease, caused by pathogenic bacteria belonging to the genus *Leptospira* (1). Large outbreaks of leptospirosis have recently occurred in many countries, especially in South-East Asian region and in South America (2). Pathogenic *Leptospira* species are invasive and have the ability to colonize and invade the renal tubules of incidental hosts like rodents, dogs, cats, cattle, pigs, and horses, presenting with variable clinical symptoms (3). Clinical manifestations are ranging from a mild febrile illness to severe disease that can include fever, headache, vomiting, diarrhea, anorexia, muscle pain and constipation (4, 5). The adhesion of pathogenic *Leptospira* to the mucous membrane of the hosts was considered to be essential during the early stage of the infection (6).

There is an urgent need to explore alternative strategies against leptospiral infections, including vaccine production. The currently available leptospiral vaccines are based on inactivated Leptospiral whole cell or membrane preparations of the pathogenic and have low efficacy. However, these vaccines stimulate antibody responses against leptospiral lipopolysaccharide, but do not provide cross-protective immunity against leptospiral serovars and generally produce only short-term immunity in domestic livestock (7, 8). So far, a large number of pathogenic and saprophytic serovars (>320) of *Leptospira* species have been described, which is a major limitation to the production of a multi-protein vaccine against multiple *Leptospira* serovars and development of immunization protocols based on whole cell or membrane preparations (9, 10). Moreover, due to lack of protection among carriage, hosts will shed the bacteria in their urine and contaminate the environments (11). Based on previous studies, several promising vaccine candidates are under evaluation. A human vaccine has been developed in China, but this vaccine is not protective in children under 14 years. A vaccine licensed for the human is still in experimental stage and therefore not approved for human usage (7, 12).

After whole genome sequencing of *Leptospira* species, a large number of leptospiral virulence factors, and surface proteins have been identified to represent new potential targets for the development of anti-leptospiral drug, vaccine and diagnostic strategies. *Leptospira* species express several outer surface proteins that may be promising candidates for the development of vaccines against leptospiral infections. LenA and LcpA proteins have been characterized as potential virulence factors. LcpA (leptospiral complement regulator-acquiring protein A) is a surface protein that binds both purified and soluble C4b binding protein (C4BP) from human sera (13). This protein is an outer membrane protein and contributes to pathogenic *Leptospira* immune evasion by binding to the complement system inhibitors (14). Based on previous studies, LcpA is found in serum resistance pathogenic *Leptospira* but not in non-pathogenic strains (15). LenA is a member of the leptospiral endostatin-like (Len) protein family, which interact with extracellular matrix (ECM), plasminogen (PLG), fibronectin, laminin, complement factor H and factor H-related protein-1 (3, 11). The genes coding for LcpA and LenA proteins are conserved among pathogenic strains of *Leptospira* (6).

To the best of our knowledge, no appropriate prophylactic studies on these leptospiral proteins have been reported. The effectiveness of these proteins in combination with LTB as an adjuvant remains to be evaluated. With the goal of discovering a new vaccine, the rLcpA, rLenA and rLTB recombinant proteins were produced in *Escherichia coli* BL21 (DE3) cells and their immunogenic potentials were considered in animal model.

## MATERIALS AND METHODS

### Cloning, expression and purification of recombinant proteins.

Genomic DNAs of *L. interrogans* serovar Copenhageni strain Fiocruz L1-130 and ETEC (Enterotoxigenic *Escherichia coli*) ATCC 35401 were extracted using DNA extraction kit (Qiagen, Germany) according to the manufacturer's instructions. The extracted DNAs were stored at −20°C until the proper time for the experiments. Amplification of *lcpA, lenA* and *ltb* genes was carried out using specific forward and reverse primers, as shown in Table 1. Following enzyme digestion of gene amplicons and vectors carried out with restriction enzymes XhoI/HindIII (for *lcpA* gene), NdeI/XhoI (for *LenA* gene) and *EcoR*I/*Hind*III (for *ltb* gene) (Fermentas, Germany), respectively. The genes amplicons were cloned into pGH vector and then finally, *lenA* gene was subcloned into pET-15b, and *lcpA* and *ltb* genes sub-cloned into pET-28a expression vectors (Novagen, USA). The expression vectors were confirmed both by restriction enzyme digestion and sequencing.

**Table 1.** List of primers used in study.

**Primer name**	**Sequence (5′ to 3′) a**	**Restriction Enzyme**	**Amplicon size (bp)**
LenA (F)	GCCCCGGGCATATGATGAATTTAAAACAAGGAAATAAAA	SmaI, NdeI	730
LenA (R)	GCCCCGGGCTCGAGCTGTTCTACACAGAGAAGATTTAG	SmaI, XhoI	
LcpA (F)	CCCGGGAAGCTTGCATGAGGAAGGAAATG	SmaI, XhoI	647
LcpA (R)	GCCCCGGGCTCGAGACAGCCAGGACCTTCG	SmaI, HindIII	
LTB (F)	GCGATATCGAATTCGCTCCTCAGTCTATTACAGAACTA	EcoRV, EcoRI	323
LTB (R)	GCGATATCCTCGAGGTTTTCCATACTGATTGCCGCAAT	EcoRV, HindIII	

a Artificial restriction sites are underlined.

The recombinant proteins were expressed in *E. coli* BL21 (DE3). Briefly, a single colony of transformed *E. coli* BL21 (DE3) containing pET-15b/*lenA* was cultured on 5 ml Luria Bertani (LB) broth containing ampicillin (100 μg/mL) and incubated at 37°C overnight (about 16 h). Next day, 200 μl of overnight culture was transferred into 5 ml of LB broth containing ampicillin (100 μg/mL) at 37°C until the OD reached to 0.6 at 600 nm. Then a final concentration of 1 mM of IPTG (isopropyl-β-D-thiogalactopyranoside) [Sigma-Aldrich] was added to bacterial cultures and the cells were grown for 4 hours at 37°C with constant shaking. The control cultures without IPTG induction were used. The bacterial cultures were centrifuged at 5,000 rpm for 5 minutes. The pellet and supernatant were transferred to separate tubes, and the cell pellets were suspended in 100 μl sodium dodecyl sulfate–polyacrylamide gel electrophoresis (SDS-PAGE) sample loading buffer and were subjected to 12.5% SDS-PAGE.

For purification of the recombinant protein, transformed *E. coli* BL21 (DE3) overexpressed in 500 ml Luria Bertani broth (Merck, Germany) containing ampicillin (100 μg/mL). The bacterial pellet was collected by centrifugation at 5000 rpm for 5 minutes and the supernatant discarded. The precipitate was dissolved in 5 ml lysis buffer (8 M urea, 100 mM NaH2PO4, 10 mMTris-HCl, pH: 8.0) and following centrifugation at 12000 rpm for 30 minutes. The supernatant loaded into Ni-NTA resin (Qiagen, Germany) column and shaken gently for 1 hour. The column was washed six times with washing buffers (urea 8 M, 6 M, 4 M, 2 M, 1 M and 0 M with pH:8.0). The recombinant protein was then eluted using elution buffer (250 mM imidazole, 50 mM NaH2PO 4.300 mM NaCl, pH:8.0). Quality of protein purification was characterized by 12.5% SDS-PAGE method. The experiments similar to the described above, carried out for transformed *E. coli* BL21 (DE3) containing pET-28a/*lcpA* and pET-28a/*ltb* with kanamycin (50 μg/mL) as a selectable marker.

### Western blotting.

Western blotting carried out to establish protein expression using His-tag monoclonal antibody conjugated with horseradish peroxidase (HRP) (Thermo Fisher Scientific, Inc., Lithuania). The recombinant proteins were electrophoresed in 12.5% polyacrylamide SDS gel and transferred onto PVDF membrane (Bio-Rad, Hercules, CA, USA) for 90 min at 4°C. Membranes were blocked in PBS containing 3% skim milk and 0.05% Tween 20 at 4°C, overnight. After blocking, membranes were washed three times with PBS containing 0.05% Tween 20 and then incubated with His-tag monoclonal antibody conjugated with HRP (at 1:1000 dilution) for 1h at 25°C. Membranes were washed three times with PBS 1X containing 0.05% Tween 20 and then treated with 3, 3′-diaminobenzidine solution (Sigma-Aldrich, USA) for about 3 min.

### Vaccine formulation.

Protein concentrations were determined using the method of Bradford. The vaccines were prepared in a proper proportion of recombinant proteins of rLenA and rLcpA with rLTB as an adjuvant. In control group, the hamsters immunized with rLTB alone. The solutions were mixed gently for 4 h at 4°C and stored at 4°C until use.

### Hamster model of leptospirosis.

Six groups consist of fifteen female Golden Syrian hamsters in each one, aged between 4 to 6 weeks, were used as the animal model. The vaccinated hamsters with the prepared protein compounds in this study, and a virulent isolate of *L. interrogans* serovar Copenhageni strain Fiocruz L1-130 were used to determine challenge dose, as described previously (16). The hamsters were immunized in the quadriceps muscle twice, with a 14-day interval between each immunization (days 0 and 14), as follows: rLenA-plus-LTB group, rLcpA-plus-LTB group, rLenA-plus-LcpA-plus-LTB group and LTB group (control). Two additional groups, Bacterin and PBS, as positive and negative control groups, respectively, accompanied with four groups which mentioned above, were considered for challenge experiments. Thirty-four days after the first immunization, all of six groups of hamsters were challenged intraperitoneally with 10^2^ cells of the virulent isolate of *Leptospira*. Before first immunization and challenge, serum of the hamsters was collected from the retro-orbital venous plexus after administration of eye anesthetic drops, and the sera were stored at −20°C, until further analyses. The hamsters were checked every day for mortality over a period of 21 days. Survivors were sacrificed 21 days post challenge. All experiments were performed in accordance with animal handling protocols approved by the Institutional Animal Care and Use Committee.

### Antibody response determination using ELISA.

The ELISA assay was performed using a commercially available kit, 20 days after second immunization on sera from the vaccinated hamsters to determine the total antibody, IgG_1_ and IgG_2_ a isotypes. Briefly, the recombinant proteins (10 μg/mL) were adsorbed in PBS 1× buffer into 96-well polystyrene plates (Greinerbio-one, Fricken-hausen, Germany) overnight at 4°C. ELISA plates were washed three times with PBST (PBS with 0.05% [v/v] Tween 20) and then blocked with PBS containing 4% skim milk for 1 h at 25°C. The plates were then washed three times using PBST, Hamster's sera were added into the wells at dilutions of 1:100 to 1:12800, and incubated for 1 h at 25°C. Following rinses, Peroxidase-conjugated anti-golden Syrian hamster IgG antibody (Rockland) was added at a 1:6,000 dilution and incubated for one hour at room temperature. The plates were washed five times with PBST, o-phenylenediamine dihydrochloride (Sigma-Aldrich) substrate solution was added and the reaction was stopped with 4N H2SO4. Finally, optical density (OD) of mixtures was read at 492 nm by ELISA Reader.

### Cytokine determination.

The concentrations of IL-17, IL-4 and IFNγ were measured by enzyme-linked immunosorbent assay. In brief, blood samples were collected from hamsters by eye bleeding after 16 h of the last immunization. Sera were separated from blood cells after coagulation at room temperature for 1 h and storage at 4°C for 1 h more with centrifugation at 12000 rpm for 10 min at room temperature. The samples were analyzed for IL-4, IL-17 and IFNγ using the sandwich ELISA assay according to the manufacturer's recommendations (eBioscience, San Diego, CA).

### Statistical analysis.

All of the Data were analyzed using SPSS software, version 22. Statistical Kruskal–Wallis test was used to compare the significant differences among different groups. The significant differences among groups were further analyzed by post hoc tests. The *p* value < 0.05 statistically was considered significant. All of the curves and charts in this study, were drawed up using GraphPad Prism 4 software.

## RESULTS

### Protein purification and western blotting analysis.

**Fig. 1.**
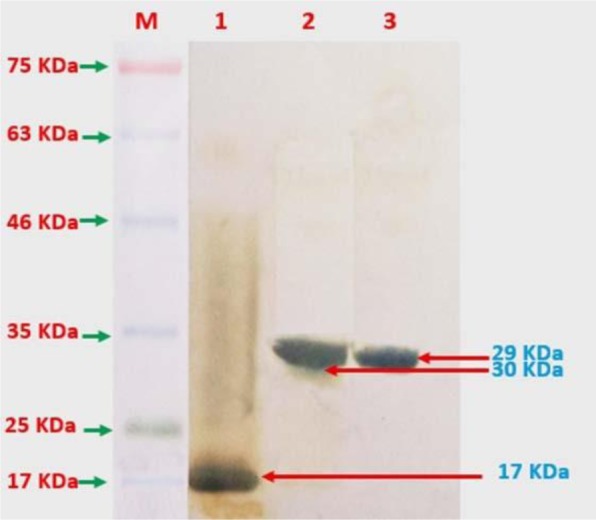
Western blotting of purified rLTB (1), rLenA (2) and rLcpA (3) recombinant proteins. M: Protein size marker

**Fig. 2.**
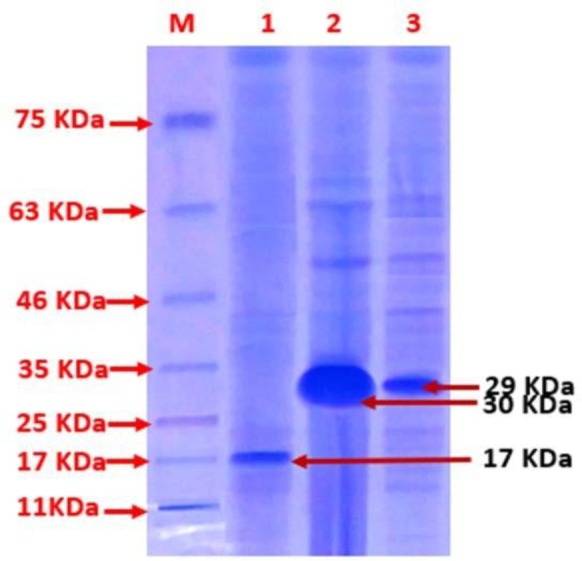
Expression of rLenA, rLcpA and rLTB recombinant proteins in 12.5% gel M: Protein size marker, 1, 2 and 3 are rLTB, rLenA and rLcpA Proteins, respectively, after induction.

After induction of the transformed *E. coli* BL21 (DE3) cells with IPTG as an inducer of gene expression, all of the recombinant proteins expressed as inclusion bodies. For purification, we used denaturing, re-naturing and eluting agents, respectively, and then yielded them as a soluble protein. The recombinant proteins were purified to near homogeneity by Ni-NTA chromatography. All three purified recombinant proteins were observed based on predicted size on the migration of SDS-PAGE gels (approximately 30 kDa for rLenA, 29 kDa for rLcpA, and 17 kDa for rLTB). Also, these proteins reacted with the anti-His monoclonal antibody in Western blotting and appeared the intended bands (Figs. 1 and 2).

### Immune response in vaccinated hamsters.

**Fig. 3.**
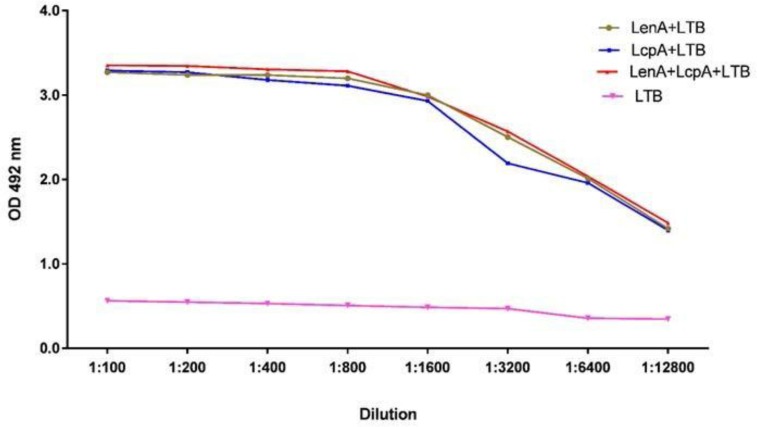
Titration of Total IgG antibodies in sera of immunized hamsters. Twenty days after the last immunization serum samples of the animals were collected and pooled. The sera were serially diluted and were coated on recombinant proteins. Different groups of animal seach consisting of 15 hamsters were administrated with either the rLenA, rLcpA and rLTB recombinant proteins. The titers of Total IgG antibodies were significantly high in hamsters (p<0.05).

**Fig. 4.**
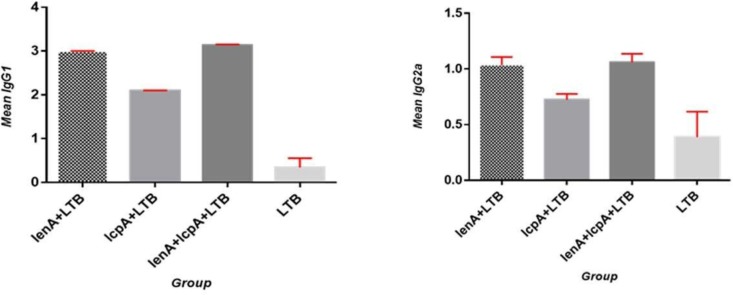
Immunoglobulin isotyping assay in sera of immunized hamsters. Twenty days after the last immunization serum samples of the animals were collected and pooled. The sera were serially diluted and were coated on recombinant proteins. Different groups of animals each consisting of 15 hamsters were administrated with either the rLenA, rLcpA and rLTB recombinant proteins. The IgG_1_ levels were higher than IgG_2_ a levels in comparison to control group (LTB). The IgG_1_ and IgG_2_ a levels against rLcpA were lower than other proteins.

Hyperimmune sera from the hamsters after the last booster injection were collected and titrated in all vaccinated groups by antigen-based ELISA. The highest OD in 492 nm (over 1:12800) of total IgG antibodies measured on the sera from the hamsters, was observed for rLenA-plus-rLcpA-plus-rLTB, rLe-nA-plus-rLTB, rLcpA-plus-rLTB, and rLTB groups, respectively. The rLenA-plus-rLcpA-plus-rLTB vaccine induced significantly higher antibody levels than the control group followed by rLenA-plus-rLTB (*p*<0.05). RLcpA-plus-rLTB and rLTB proteins did not induce any significant antibody response in vaccinated hamsters (*p*>0.05) (Fig. 3). The types of immune responses to the recombinant proteins were further examined by measuring the levels of IgG_1_ and IgG_2_ a subclasses. The hamsters inoculated with recombinant proteins had significantly higher IgG_1_ titers than IgG_2_ a in comparison to control groups with a statistical significance (*p*<0.05) (Fig. 4).

### Efficacy of vaccine preparations.

**Fig. 5.**
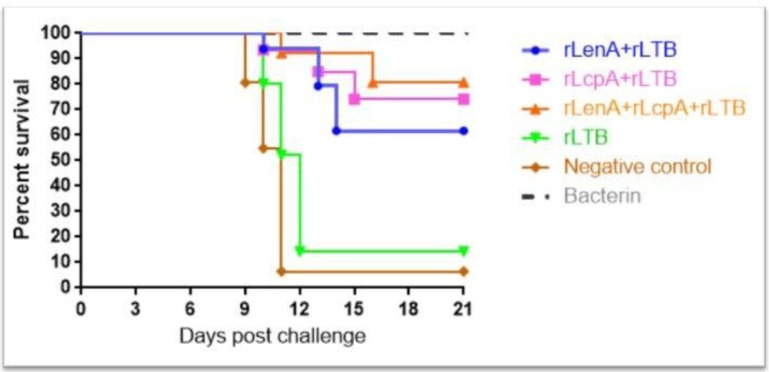
Cumulative survival rates of hamsters challenged with *L. interrogans* serovar Copenhageni strain Fiocruz L1-130. The hamsters were intraperitoneally infected with 10^2^ cells of *L. interrogans*. The Survival rates of each group were checked for 21 days. There were statistically significant differences (*p*<0.05) between immunized hamsters and control groups.

The protective efficacy of the rLenA and rLcpA in combination with rLTB vaccine preparations were evaluated. The hamsters which were immunized with the mentioned protein compositions and also Bacterin and PBS (control) groups, were challenged with 10^2^ cells of *L. interrogans* serovar Copenhageni strain Fiocruz L1-130, intraperitoneally injection. The hamsters immunized with the rLenA-plus-rLcpA-plus-rLTB, rLenA-plus-rLTB and rLcpA-plus-rLTB, showed statistically significant improvement (*p*<0.05) in survival rate after bacterial challenge (Fig. 5). The median survival times for hamsters immunized with the recombinant proteins were significantly longer than hamsters received PBS alone (*p*<0.05). The highest survival rate was observed in rLenA-plus-rLcpA-plus-rLTB group.

### Cytokine expression profile in immunized hamsters.

**Fig. 6.**
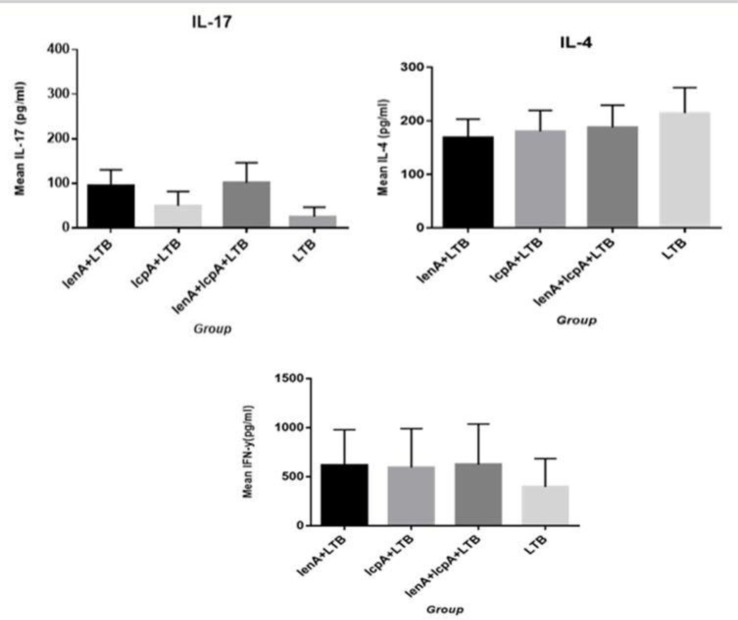
Levels of IL-4, IL-17 and IFNγ cytokines (pg/mL) in hamsters. The levels of IL-17 was very low compared to two other cytokines and did not show statistical differences with group that received rLTB alone (*p*>0.05). There were no statistically significant differences (*p*>0.05) between the groups.

A significant amount of IL-17, IL-4 and IFNγ cytokines was produced in immunized hamsters (Fig. 6). There were not statistically significant differences (*p*>0.05) between the groups. The levels of IFNγ in the immunized hamsters with rLenA-plus-rLcpA-plus-rLTB, rLenA-plus-rLTB and rLc-pA-plus-rLTB did not show statistically significant difference (*p*>0.05) with group received rLTB alone.

## DISCUSSION

*Leptospira* has a wide variety of mechanisms that enable it to evade the host immune system and enhance the severity of the infection. The identification of protective antigens is needed to facilitate vaccine-based prophylactic approaches. Some of identified proteins contribute to the ability of the bacteria to adhere, invade and colonize onto the host tissues. Towards controlling the increasing problem of leptospirosis, for the first time we evaluated, LenA and LcpA proteins from *L. interrogans* serovar Copenhageni strain Fiocruz L1-130 in combination with *E. coli* heat-labile enterotoxin, B subunit (LTB) as potential candidates for vaccine production against Leptospiral infections.

The available leptospiral vaccines are based on inactivated whole-cell or membrane preparation (18). However, these vaccines stimulate antibody responses, fail to induce immune memory and consequently require booster injections. Moreover, these vaccines bear considerable side-effects and do not provide cross-protective immunity against serovars (19, 20). Leptospiral immunoglobulin-like (Lig) proteins are probably the best vaccine candidates, as they demonstrated 90–100% of protection in immunized hamsters that survived lethal bacterial challenge. However, these proteins are not conserved amongst all pathogenic strains and sterilizing immunity has not yet been achieved (21, 22), highlighting the need for new conserved antigens for development of a better vaccine. In the present study we evaluated two protein LenA and LcpA which are highly conserved among all pathogenic leptospires and could potentially confer cross-protection. However, these proteins showed lower protection (60–80%) in comparison to Lig proteins.

Our results showed that the rLenA and rLcpA proteins in combination with rLTB induce high IgG titers in hamsters. Similar studies demonstrated that cell surface protein Lig could produce high IgG titers in hamsters (18). In another similar study conducted by Oliveira et al., recombinant OmpL 37 induced a strong IgG response in hamsters inoculated with different vaccine formulations (21). In this study, recombinant proteins were emulsified with rLTB as adjuvant, which generated high titers of anti-LcpA and anti-LenA. A comparable study that used LTB along with recombinant protein LipL 32 showed significantly high titers against the leptospiral antigen (23).

In this study, the plasma level of IFN gamma produced in the hamsters immunized by rLenA and rL-cpA recombinant proteins was higher than IL-4 and IL-17. The high-level production of this cytokine in response to recombinant proteins may be due to the stimulation of Th-1 lymphocytes. Potent vaccine protection against leptospirosis has attributed to induction of Th-1 immune responses (23, 24). Several studies have reported that Th-1 and other components of innate immune systems are essential for bacterial clearance in animal models (24, 25).

Results of present study revealed that the rLenA and rLcpA recombinant proteins efficiently stimulated cell mediated immunity. Our results predict that these proteins in combination with LTB recombinant have the potential for broad protective coverage against leptospiral infection. Both rLenA and rLcpA proteins can be used as candidate for vaccine development along with rLTB for better efficacy. Further investigation on the role of rLenA and rLcpAin ligand binding and epitope mapping delineation might be necessary to further improvement of the leptospiral vaccines. Studies tailored towards identifying novel potential vaccine candidates against leptospiral infections are essential and still need.
